# Supplementary material for: The in vivo transcriptome of Schistosoma mansoni in the prominent vector species Biomphalaria pfeifferi with supporting observations from Biomphalaria glabrata
Source: PLoS Negl Trop Dis. 2019 Sep 30;13(9):e0007013. doi: 10.1371/journal.pntd.0007013 (PMC6797213; doi:10.1371/journal.pntd.0007013)
Supplement: S1 Table — (DOCX) [file pntd.0007013.s001.docx]

| **Filtered reads used in *S. mansoni de novo* assemblies** | **222,593,797** |
| --- | --- |
| ***De novo* assembled contigs** |  |
| Trinity *de novo* Illumina | 18,860 |
| Genome-guided Trinity *de novo* Illumina | 26,993 |
| Genome-guided Trinity *de novo* 454 | 5,767 |
| ***S. mansoni* transcripts** | **23,602** |
| % GC | 35.40% |
| N_50_ | 1,412 |
| Median transcript length | 479 |
| Average transcript length | 841.92 |
| Transcripts ≥500nt | 11,419 (48.4%) |
