# Supplementary figures and images for: The in vivo transcriptome of Schistosoma mansoni in the prominent vector species Biomphalaria pfeifferi with supporting observations from Biomphalaria glabrata
Source: PLoS Negl Trop Dis. 2019 Sep 30;13(9):e0007013. doi: 10.1371/journal.pntd.0007013 (PMC6797213; doi:10.1371/journal.pntd.0007013)

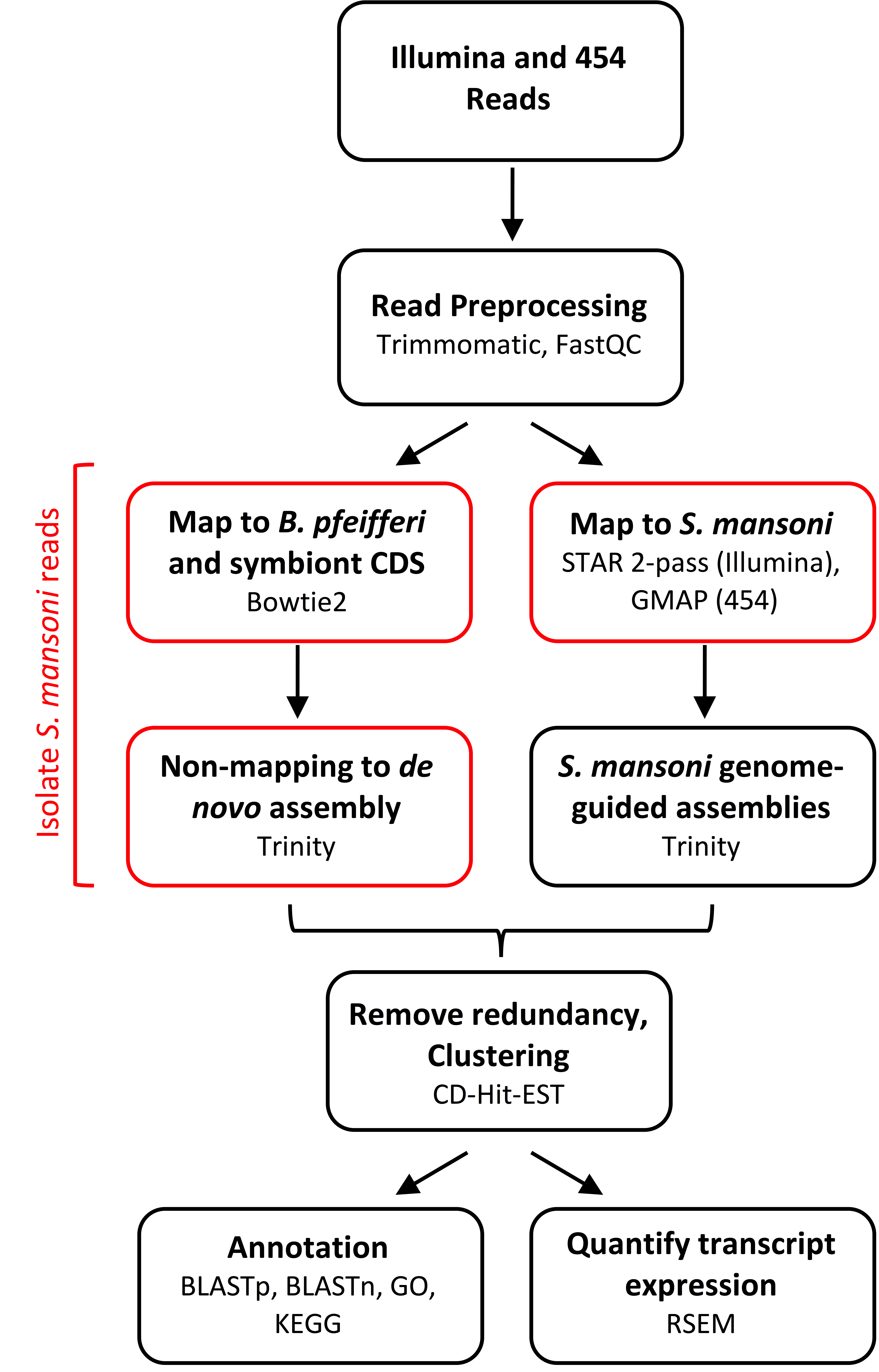

Supplement: S1 Fig — (TIFF) [file pntd.0007013.s003.tiff]

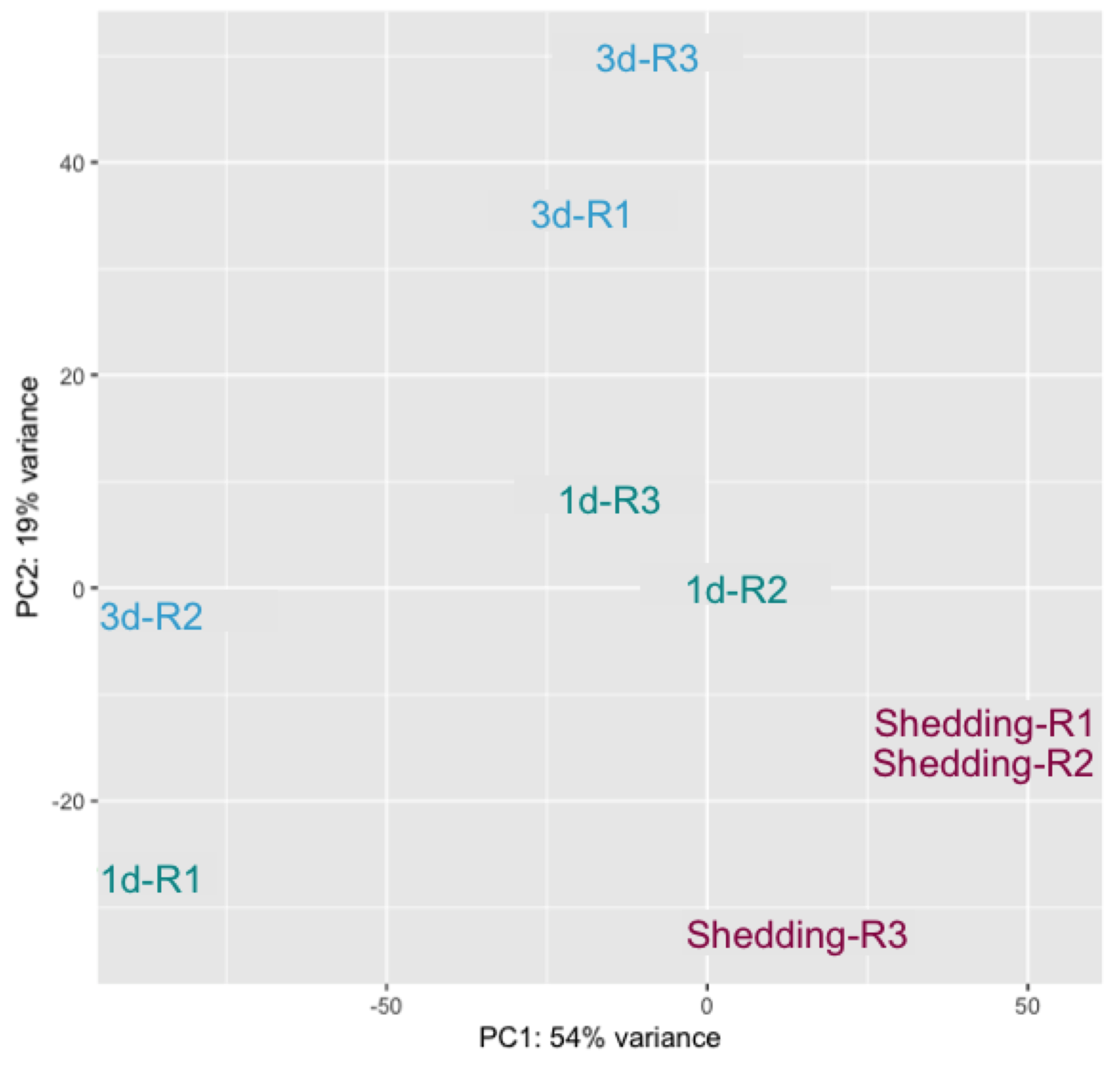

Supplement: S3 Fig — (TIFF) [file pntd.0007013.s005.tiff]

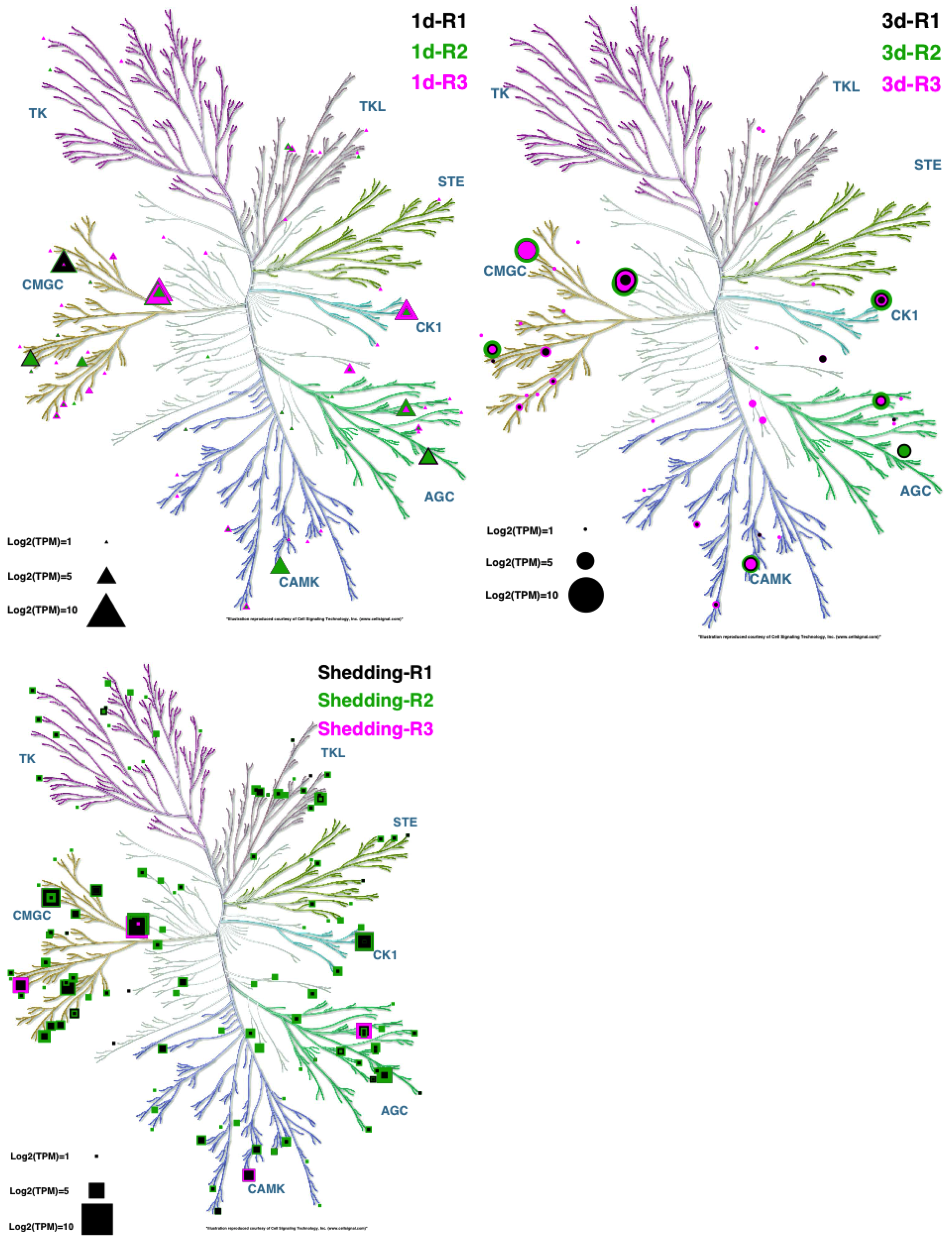

Supplement: S5 Fig — TK: phosphorylate tyrosine residues; TKL: “tyrosine kinase-like” serine-threonine protein kinases; STE: mostly protein kinases involved in MAP (mitogen-activated protein) kinase cascades; CK1: casein kinases; AGC: cyclic-nucleotide-dependent family (PKA, PKG), PKC, and relatives; CAMK: calcium/calmodulin modulation activity; CMGC: cyclin-dependent kinases, MAP kinases, glycogen synthase kinases, and CDK-like kinases. The figure was generated using KinomeRender. (TIFF) [file pntd.0007013.s007.tiff]

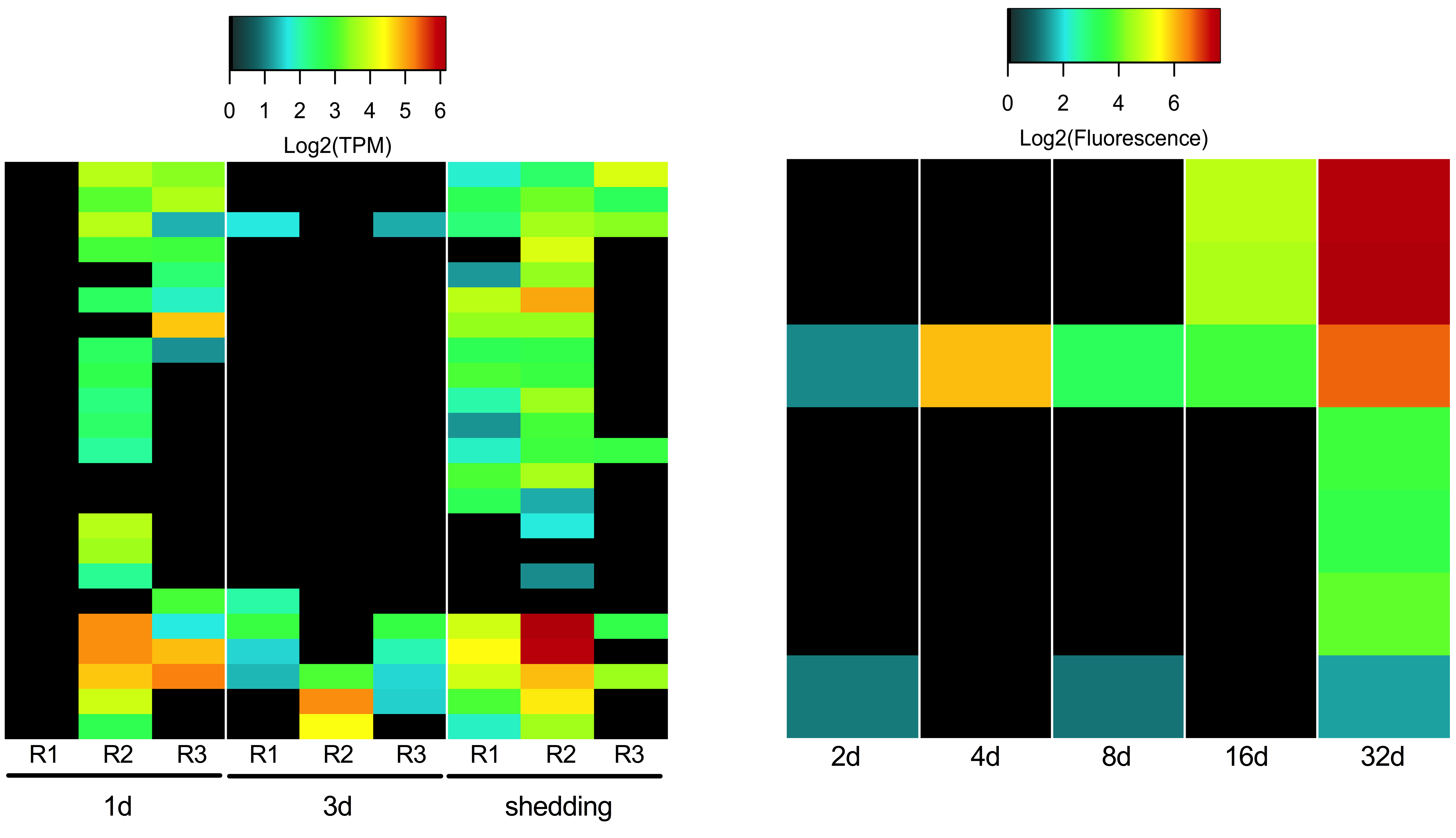

Supplement: S7 Fig — (TIFF) [file pntd.0007013.s009.tiff]
